# Controlling nutritional status score predicts postoperative complications after hip fracture surgery

**DOI:** 10.1186/s12877-020-01643-3

**Published:** 2020-07-13

**Authors:** Toshio Yagi, Yusuke Oshita, Ichiro Okano, Takuma Kuroda, Koji Ishikawa, Takashi Nagai, Katsunori Inagaki

**Affiliations:** 1 Department of Orthopedic Surgery, Showa University School of Medicine, 1-5-8 Hatanodai Shinagawa-ku, Tokyo, 142-8555 Japan; 2 Department of Orthopedics, Showa University Northern Yokohama Hospital, 35-1 Chigasaki-cho, Tsuzuki-ku, Yokohama, 224-8503 Japan; 3 Department of Orthopedic Surgery, Ohta-Nisihinouchi Hospital, 2-5-20 Nishinouchi, Koriyama, 963-8558 Japan

**Keywords:** Hip fracture, Nutrition, Controlling nutritional status, CONUT score, Postoperative complication, Elderly patient

## Abstract

**Background:**

Controlling Nutritional Status (CONUT) score is calculated using laboratory values, including serum albumin, total cholesterol concentration, and total lymphocyte count; it is reportedly valuable for making nutritional assessments. One advantage of CONUT score over other nutritional assessments is that it can be calculated retrospectively using only objective laboratory values. Studies demonstrated that CONUT score was a useful tool for predicting prognosis and complications in various surgical conditions. Nevertheless, few studies utilized the score as a potential predictive marker for postoperative complications among hip fracture patients. The purpose of this study was to determine the association between CONUT score and postoperative complications in hip fracture patients.

**Methods:**

We retrospectively reviewed 211 elderly patients who underwent hip fracture surgery at a single institution from 2013 to 2018. CONUT score was calculated using preoperative routine laboratory tests for serum albumin, total cholesterol concentration, and total lymphocyte count. As potential confounders, we extracted data such as patient age, sex, fracture type, and general conditions/comorbidities, as defined by the American Society of Anesthesiologists Physical Status (ASA-PS) classification and the Charlson Comorbidity Index (CCI). Postoperative complications were defined as a Clavien-Dindo classification of 1 or more. Simple and multivaribale logistic regression analyses were performed to assess the incidence of postoperative complications as the outcome measures.

**Results:**

The mean age [IQR] was 86 [80–90], and 80.1% of the reviewed patients were female. Based on the CONUT scores, 78.7% of hip fracture patients were classified as malnourished; 18% experienced postoperative complications. Simple analyses revealed significant risk factors for postoperative complications, including age, the ASA-PS, the CCI, and the CONUT score. Multivariable analysis found that CONUT score was the independent risk factor for postoperative complications (odd ratio = 1.21, 95% confidence interval = 1.01–1.45, *p* = 0.04).

**Conclusions:**

Preoperative CONUT scores are independently associated with the incidence of postoperative complications. CONUT score can be used for risk assessment in hip fracture patients to predict early postoperative complications.

## Background

Currently osteoporosis is one of the most prevalent diseases in many developed countries because of an aging population. The number of patients with osteoporotic fractures is expected to increase rapidly [1, 2]. Osteoporotic hip fracture has the greatest impacts on patients’ function and life expectancy among all fragility fractures. Elderly patients who sustain a hip fracture are more likely to sustain postoperative medical and surgical complications such as pneumonia, heart failure, and urinary tract infections than other age groups of surgical patients [3–5].

Poor nutritional status is one of the major contributing factors for postoperative complications in many types of surgery [6, 7]. Several nutritional assessment systems have been introduced. The Subjective Global Assessment (SGA) enables the clinician to determine the nutritional status of the patient; it has been widely used as a global assessment of nutritional status for various surgical conditions [8]. However, one major drawback of these assessment systems is that they include one or more measurements that inherently depend on a degree of subjectivity. Another major drawback is that these subjective measurements cannot be conducted retrospectively. In other words, if one of these assessments is not conducted prior to surgery, it is impossible to evaluate later on the surgical risk associated with nutritional status with these assessment systems.

Controlling Nutritional Status (CONUT) was first introduced by Ignacio et al. [9]; it requires only common laboratory tests that are often performed routinely in presurgical assessments and/or periodical health assessment examination by general practitioners. CONUT allows examiner-independent and retrospective evaluations for nutritional status. Previous studies demonstrated that CONUT highly correlated with SGA [10] and was useful to stratify the risk for postoperative complication among patients undergoing surgery for gastrointestinal and hepatopancreatobiliary cancers [11]. Despite these benefits of CONUT, few studies have addressed osteoporotic hip fractures. Therefore, the purpose of this study was to investigate the association between nutritional status evaluated using CONUT and postoperative complications.

## Methods

### Study population

Institutional ethical committee approval was obtained for this study (No10–10). We retrospectively reviewed clinical data of consecutive patients with hip fracture between 2013 and 2018 at a single community-based hospital. We excluded young patients (< 50 y), those receiving conservative treatment, and those who suffered high-energy trauma or pathological fracture. We recorded potential confounders, including age, sex, fracture type, blood hemoglobin concentration, days until return to long-term residence, discharge destination, American Society of Anesthesiologists physical status (ASA-PS) classification, and Charlson Comorbidity Index (CCI) [12].

### CONUT score and complication assessment

Preoperative blood samples were taken on the day of admission or the following day in patients admitted outside of daily working hours. The preoperative CONUT scores were calculated using the results of three laboratory tests; serum albumin concertation, lymphocyte count, and total cholesterol concentration (Table 1).

**Table 1:** Assessment of the nutritional status using CONUT

Parameter	Malnutrition status			
	None	Light	Moderate	Severe
Serum albumin (g/dL)	≧3.5	3.0–3.49	2.5–2.99	<2.5
**Albumin score**	**0**	**2**	**4**	**6**
Total lymphocyte count (/mm^3^)	≧1600	1200–1599	800–1199	<800
**Lymphocyte score**	**0**	**1**	**2**	**3**
Total cholesterol (mg/dl)	≧180	140–179	100–139	<100
**Cholesterol score**	**0**	**1**	**2**	**3**
**Total score**	**0–1**	**2–4**	**5–8**	**9–12**

*CONUT* Controlling Nutritional Status

As the primary outcome measure, postoperative complications were documented utilizing the Clavien–Dindo (CD) classification for surgical complications [13]. The CD classification is a five-grade assessment system for general postoperative complications; Grade I is defined by deviation from the normal postoperative course, including noninfectious diarrhea, or transient elevation of serum creatinine. Grade II is defined by conditions requiring medical therapy without surgical treatment, including pneumonia treated with antibiotics in the ward, or urinary tract infection requiring antibiotics. Grade III is defined by surgical, endoscopic or radiological intervention, including wound infection requiring debridement, or bradyarrhythmia requiring pacemaker implantation under local anesthesia. Grade IV is defined by life-threatening complications requiring intensive care, including respiratory failure requiring intubation, or ischemic stroke. Grade V is defined by death due to complications. We defined a postoperative complication as any type of postoperative complication with CD grade ≥ 1 occurring between the time of surgery until 30 days after the primary procedure.

### Statistical analysis

Statistical analyses were performed utilizing the Chi-squared test for categorical variable comparisons, and logistic regression test for simple and multivariable analyses. The statistical significance was set at *p* < 0.05. All the analyses were carried out using R software (R for 3.4.1 GUI 1.64) [14].

## Results

### Patient demographics and clinical characteristics

**Fig. 1:**
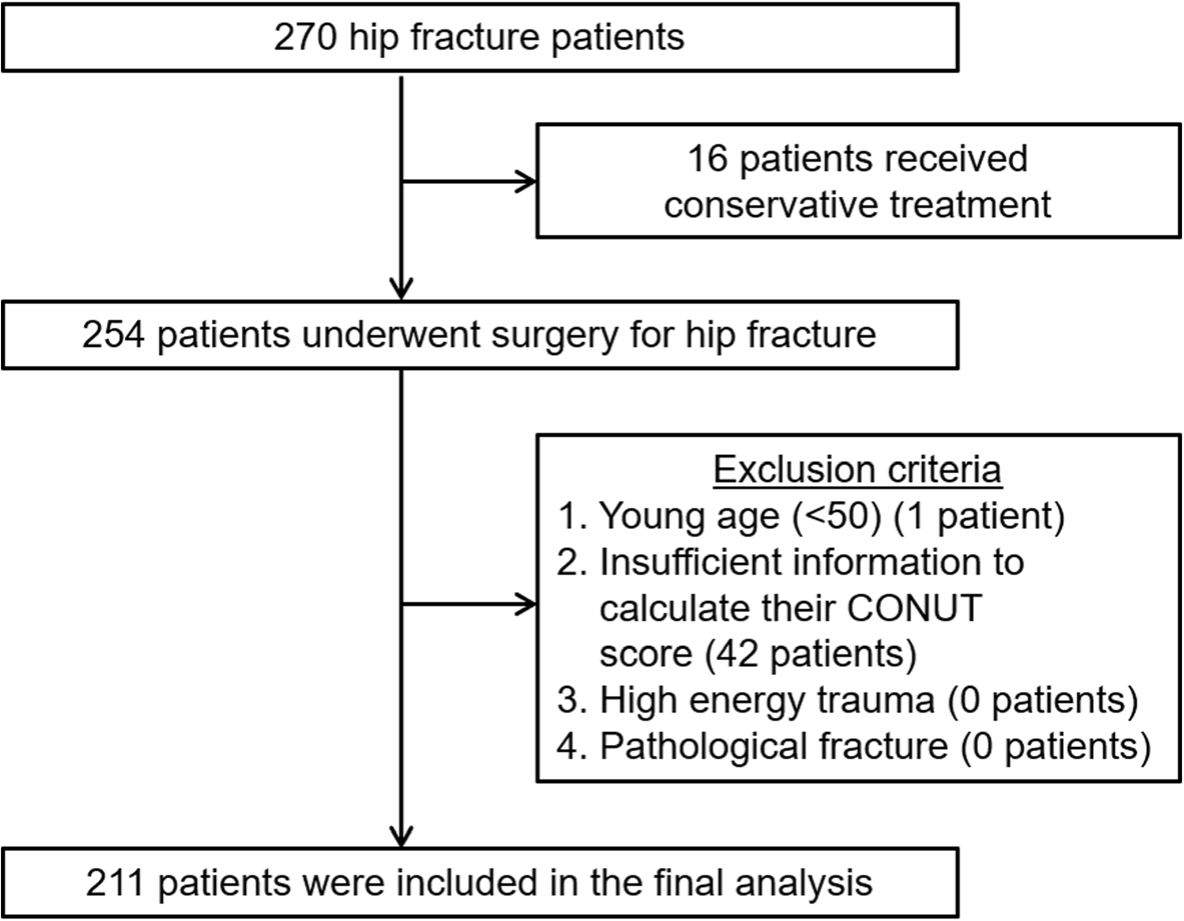
Flow diagram of inclusion criteria

A total of 211 patients were identified (Fig. 1). The median age [IQR] was 86 [80–90] years; 80.1% of the patients were female; 66.8% of patients were ASA-PS class 2, and the median CCI score [IQR] was 1.0 [0–2]; 78.7% of the patients were malnourished, as defined by a CONUT score > 1. Of these 211 patients, 39 (18.5%) experienced at least one postoperative complication (14 urinary tract infections, six heart failures, four surgical site infections, four re-fractures, three pneumonias, three arrhythmias, three cases of enteritis, and 10 others) (Tables 2 and 3). The mean duration between the hospital admission to final discharge to long-term residences, such as home or a nursing care facility, was 29.5 [21–41] days in patients with no postoperative complications and 43 [31–52.5] days in patients with postoperative complications (*p* = 0.03). Thirty-day mortality rates were 0.95%. The percentage of patients originally living at home who were discharged home was 38.4%. Demographics and clinical characteristics of the patients are summarized in Table 4.

**Table 2:** Postoperative complications

Postoperative Complication (%)	
Urinary tract infection	14 (29)
Heart failure	6 (12)
Surgical site infection	4 (8)
Re-fracture	4 (8)
Pneumonia	3 (6)
Arrhythmia	3 (6)
Enteritis	3 (6)
Liver dysfunction	2 (4)
Implant failure	2 (4)
Acute myocardial infraction	1 (2)
Venous thromboembolism	1 (2)
Asthma	1 (2)
Gastrointestinal bleeding	1 (2)
Heterotopic ossification	1 (2)
Pseudo-gout	1 (2)
Renal dysfunction	1 (2)
Cholangitis	1 (2)

**Table 3:** Clavien-Dindo classification grade

Clavien-Dindo classification grade (%)	
I	2 (4.7)
II	23 (53.5)
III	15 (34.9)
IV	1 (2.3)
V	2 (4.7)

Grade I is defined by deviation from the normal postoperative course

Grade II is defined by conditions requiring medical therapy without surgical treatment Grade III is defined by surgical, endoscopic or radiological intervention

Grade IV is defined by life-threatening complications requiring intensive care

Grade V is defined by death due to complications

**Table 4:** Demographics, patient characteristics and the results of simple logistic regression analysis

Demographic characteristics		Postoperative complications			
	All patients (*N* = 211)	No (*N* = 172)	Yes (*N* = 39)	OR (95% CI)	*p*-value
Age (Med [IQR])	86 [80–90]	85 [78.8–90]	88 [85.5–91.5]	**1.06 (1.01–1.11)**	**0.01**
Sex (%)					
Women	169 (80.1)	137 (79.7)	32 (82.1)	ref	Ref
Men	42 (19.9)	35 (20.3)	7 (17.9)	0.86 (0.35–2.1)	0.74
Fracture type (%)					
Intra-articular	84 (39.8)	68 (39.5)	16 (41.0)	ref	Ref
Extra-articular	122 (57.8)	101 (58.7)	21 (53.8)	0.88 (0.43–1.81)	0.73
Subtrochanteric	5 (2.4)	3 (1.7)	2 (5.1)	2.83 (0.44–18.4)	0.28
Days until return to long-term residence (Med [IQR])	31 [23–43]	29.5 [21–41]	43 [31–52.5]	**1.02 (1.00–1.04)**	**0.03**
Discharge destination (%)					
Home	85 (40.2)	76 (89.4)	9 (10.6)	ref	Ref
Nursing care institution	126 (59.7)	96 (76.2)	30 (23.8)	**2.4 (1.16–4.92)**	**0.02**
ASA class (%)					
1	11 (5.2)	11 (6.4)	0	ref	Ref
2	141 (66.8)	119 (69.2)	22 (56.4)	(PS1–2)	
3	58 (27.5)	42 (24.4)	16 (41.0)	2.39 (1.16–4.92)	**0.02**
4	1 (0.5)	0 0.0)	1 (2.6)	(PS3–4)	
CCI (%)	1.0 [0–2]	1.0 [0–2]	1.0 [1–2]		
0	61 (28.9)	57 (33.1)	4 (10.3)	ref	Ref
1	71 (33.6)	49 (28.5)	22 (56.4)	6.4 (2.06–19.8)	**0.01**
2<	79 (37.4)	66 (38.4)	13 (33.3)	2.81 (0.86–9.09)	0.09
Hemoglobin (g/dl) (Mean (SD))	11.8 (1.9)	11.0 (1.8)	12.0 (1.8)	**0.75 (0.62–0.91)**	**0.004**
CONUT (med [IQR])	3.1 [0.00–11.00]	3.0 [0.00–11.00]	4.00 [0.00–10.00]	**1.30 (1.10–1.54)**	**0.002**
Normal	45 (21.3)	33 (73.3)	12 (26.7)		
Light	115 (54.5)	91 (79.1)	24 (20.9)		
Moderate	49 (23.2)	36 (73.5)	13 (26.5)		
Severe	2 (0.9)	0	2 (100)		

*OR* Odds Ratio

*CI* Confidence Interval

*IQR* Interquartile Range

*ref* Reference

*ASA-PS* American Society of Anesthesiologists Physical Status

*CCI* Charlson Comorbidity Index

*SD* Standard Deviation

*CONUT* Controlling Nutritional Status

### Risk factors for postoperative complications

To identify potential risk factors for postoperative complications, simple analyses were carried out. Preoperative age (*p* = 0.01), ASA-PS (*p* = 0.02), Hemoglobin (*p* = 0.004) and CONUT score (*p* = 0.002) were significant risk factors for postoperative complications (Table 4). Multivariable logistic regression analysis, adjusted for age, ASA-PS and CCI, demonstrated that CONUT score was the independent risk factor for postoperative complication (OR = 1.21, 95% CI = 1.01–1.45, *p* = 0.04) (Table 5).

**Table 5:** Multivariable logistic regression analysis

Parameters	Multivariable Logistic Regression		
	OR	95% CI	*p*-value
Age	1.05	0.997–1.10	0.067
ASA-PS	1.94	0.903–4.15	0.089
CCI	1.13	0.822–1.56	0.445
CONUT score	1.21	1.01–1.45	**0.040**

Independent variables: Age, ASA-PS, CCI, CONUT score

Dependent variable: Postoperative complication

*CI* Confidence interval

*ASA-PS* American Society of Anesthesiologists Physical Status

*CCI* Charlson Comorbidity Index

*CONUT* Controlling Nutritional Status

## Discussion

We found that the CONUT score was an independent factor for postoperative complications in our population. Of 211 patients, 39 (18%) had postoperative complications, consistent with results of other studies reporting 19–20% overall complication rates [3, 4].

CONUT was developed by Ignacio de Uli’Barri et al. in 2005 as a screening tool for malnutrition among general hospitalized patients [9]. A recent systematic review and meta-analysis showed that preoperative CONUT score was associated with increased risk of mortality and complications in surgical patients with gastrointestinal and hepatopancreatobiliary cancers [11]. Another retrospective study found that CONUT score was useful for predicting long-term mortality in hospitalized patients with heart failure [15]. Despite abundant evidence showing the usefulness of CONUT score as the index of malnutrition for short and long-term clinical outcomes of various conditions, we found only one study which utilized CONUT score as a predictive marker for hip fracture patients. The results showed that CONUT score predicted the 180-day mortality well in hip fracture patients: the survivors’ mean CONUT score was 4 ± 2, whereas the non-survivors’ CONUT score was 6 ± 3 [16]. The study did not include the association between CONUT and postoperative complications, whereas we provided novel information about the association between CONUT score and postoperative complications.

There have been several retrospective studies investigating associations between malnutrition and clinical outcomes in hip fracture patients. These studies utilized serum albumin concentration as the index of malnutrition and showed significant associations between hypoalbuminemia (albumin < 3.5 g/dL) and increased postoperative complications and mortality [17–19]. Another study found that the group with low albumin and low lymphocyte counts at the time of hospital admission had a higher 12-month mortality rate than did the group with a normal albumin concentration [20].

Other studies utilized clinical assessments based on physical examination and post medical history, in which certain elements were associated with malnutrition, as prognostic markers for hip fracture patients. Ahman et al. reported that ASA-PS, a system for assessing the fitness of patients for surgery, was an independent predictor of one-year mortality after hip surgery [21]. Another study demonstrated that CCI, based on the number of index comorbidities, correlated with short-and long-term mortality rates after surgical treatment of hip fracture [22]. In our study, only CONUT score showed statistical significance in the multivariate analysis, whereas ASA-PS and CCI were not independent risk factors for complications after hip fracture. One drawback of ASA-PS is that this system inherently depends on the subjectivity of examiners. One study showed that the interrater reliability of ASA-PS was only moderate, with kappa values around 0.60 [23]. ASA-PS includes six categories; however, categories 5 (moribund) and 6 (organ donor) are generally not used for fracture patients, and category 1 (healthy) patients are rare among patients with fragility hip fractures. For these reasons, in real patients with hip fractures, only three categories can be counted. This could be another shortcoming of ASA-PS for detailed risk stratification of hip fracture patients. CCI has less room for subjectivity, because it is based on the number of comorbidities. Nevertheless, one major drawback of CCI is that the severity of each disease is only included in a limited number of items, including diabetes, liver disease, and cancer. For example, heart failure is always allotted one point, regardless of its severity. These points might have contributed to our results and might highlight the importance of objective quantitative nutritional assessment for prediction of postoperative complications after hip fracture surgery.

Compared with other nutritional specific assessments, including SGA, Mini Nutritional Assessment-Short Form (MNA-SF) [24], and Malnutrition Screening Tool (MST) [25], CONUT has unique advantages. First, CONUT score can be calculated only with objective laboratory values. This means that CONUT does not require detailed history-taking. All other nutritional assessments include factors based on past medical history or recent food intake. Hip fracture patients often have dementia [26], making it extremely difficult to collect accurate information using medical interviews. Furthermore, CONUT scores can be retrospectively calculated when needed without physical examinations. This feature is especially useful in the research setting or when treating referred patients. By contrast, it is impossible to calculate the SGA score when certain examinations are not conducted at the time of admission. It was reported that the SGA had a low inter-rater reliability (13%), despite the fact that kappa increased as examiners gained experience [27]. By contrast, CONUT score is automatically calculated using objective values and is completely free from rater reliability issues. Regarding other measurement systems, Bel et al. reported that the specificity of MNA-SF for malnutrition defined by ICD-10-code was not high (49%). MST demonstrated low sensitivity (60%) for malnutrition in hip fracture patients [28]. By contrast, CONUT score showed excellent diagnostic ability with area under the curve as 0.86 for clinically diagnosed malnutrition [10]. In certain conditions, CONUT score should be interpreted with caution, as it may be affected by nutritional status, immune status, inflammation, metabolic diseases, and dehydration. For example, in patients with acute bacteremia or certain hematological conditions, the lymphocyte count may be disproportionally low. In such cases, a high CONUT score does not necessarily indicate malnutrition.

The present study has some limitations. First, this was a retrospective study conducted at a single institution, and the number of cases was limited. Additionally, because of the retrospective nature of this study and the lack of specific protocols to detect the complications, the incidences of certain complications, such as delirium, were likely underestimated and/or not well-differentiated from other complications. One report demonstrated that delirium was misdiagnosed in over 60% of cases if the diagnosis was made solely by physicians’ impression [29]. To address the accurate estimation of complication rates, standardized diagnostic protocols are warranted in future studies. We investigated the associations between CONUT score and postoperative complications, but did not compare CONUT score to postoperative mobility status or rehabilitation outcome, due to the data availability and our study population including only surgically treated patients. Thus, the results cannot be directly applied to patients undergoing conservative treatment. Furthermore, blood samples were taken at time of admission or the next day; therefore, blood sampling conditions were not consistent, and the performance status was only evaluated with ASA-PS, which is relatively simple. Fourth, there were no direct comparisons with other nutritional indicators such as SGA, MNA-SF, or MST, because of the retrospective nature of this study. This should be addressed in future prospective studies. Additionally, we evaluated complications at 30 days postoperatively, making it impossible to draw any conclusions about long-term prognosis. Studies with longer follow-up are warranted to address this issue. Finally, this study was observational and did not include any results of interventions for malnutrition that might improve the prognosis of hip fracture patient population. Espauella et al. showed that hip fracture patients who received nutritional supplementation had lower complications and hospital length of stay [30]. Another multicenter prospective cohort study showed that abdominal surgical patients who received nutritional support had a lower complication rate than did the control group (25.6% versus 50.6%) [31]. In future studies, nutritional interventions based on CONUT assessments should be evaluated to improve the outcomes.

## Conclusions

The CONUT score was an independent predictor of postoperative complications after adjustment for ASA-PS and CCI. These findings suggest that the preoperative CONUT score is a useful screening tool for nutritional assessment hip fracture patients to predict early postoperative complications. CONUT can be a useful tool for nutritional assessment in future hip fracture studies and daily practice.

## Data Availability

Data that support the findings of this study are available from the corresponding author on reasonable request.

## Abbreviations

CONUT: Controlling Nutritional Status
SGA: Subjective Global Assessment
ASA-PS: American Society of Anesthesiologists physical status
CCI: Charlson Comorbidity Index
CD: Clavien-Dindo
SD: Standard deviation
IQR: Interquartile Range
OR: Odds ratio
CI: Confidence interval
ref.: Reference
MNA-SF: Mini Nutritional Assessment-Short Form
MST: Malnutrition Screening Tool
ICD: International Statistical Classification of Diseases and Related Health Problems
